# Cross-Domain Active Learning for Electronic Nose Drift Compensation

**DOI:** 10.3390/mi13081260

**Published:** 2022-08-05

**Authors:** Fangyu Sun, Ruihong Sun, Jia Yan

**Affiliations:** 1 WESTA College, Southwest University, Chongqing 400715, China; 2 College of Artificial Intelligence, Southwest University, Chongqing 400715, China; 3 Brain-Inspired Computing and Intelligent Control of Chongqing Key Laboratory, Southwest University, Chongqing 400715, China

**Keywords:** electronic nose, active learning, cross-domain learning, drift compensation

## Abstract

The problem of drift in the electronic nose (E-nose) is an important factor in the distortion of data. The existing active learning methods do not take into account the misalignment of the data feature distribution between different domains due to drift when selecting samples. For this, we proposed a cross-domain active learning (CDAL) method based on the Hellinger distance (HD) and maximum mean difference (MMD). In this framework, we weighted the HD with the MMD as a criterion for sample selection, which can reflect as much drift information as possible with as few labeled samples as possible. Overall, the CDAL framework has the following advantages: (1) CDAL combines active learning and domain adaptation to better assess the interdomain distribution differences and the amount of information contained in the selected samples. (2) The introduction of a Gaussian kernel function mapping aligns the data distribution between domains as closely as possible. (3) The combination of active learning and domain adaptation can significantly suppress the effects of time drift caused by sensor ageing, thus improving the detection accuracy of the electronic nose system for data collected at different times. The results showed that the proposed CDAL method has a better drift compensation effect compared with several recent methodological frameworks.

## 1. Introduction

Electronic noses (E-noses) are sensor intelligence systems in the field of artificial olfaction that mimic the olfactory system of mammals to identify or measure gas samples. A complete E-nose system typically consists of three components: a gas sensor array, a data pre-processing unit, and a pattern recognition algorithm module. An E-nose has sensitivity to complex gas mixtures/compounds as well as analytical capabilities, thus allowing it to accurately identify complex gas samples. With the development of artificial olfaction, E-noses are also becoming increasingly important in fields such as [1], environmental monitoring [2], biomedical detection [3], and medical diagnostics [4]. However, drift with the sensor aging, which occurs over time, is still unavoidable due to the sensitivity change in the manufacturing and use process of gas sensors [5,6]. Although the drift of the E-nose sensors is progressively larger over time, this drift is nonlinear, irregular, and unpredictable and is not directly measurable through time variation. In addition, the effect of sensor aging on different types of gases is also different, which leads to the difference of sensitivity change of the sensors to different gases, and consequently results in the situation that the E-nose data do not have consistent drift trend. As mentioned above, sensor drift in the E-nose system is very complex and difficult to measure directly. It can significantly reduce the recognition accuracy of gas sensors and cause many problems in practical applications. Therefore, a suitable and effective method of drift compensation for E-noses is required to address this common problem in the field of artificial olfaction.

Due to drift, it will lead to an inconsistent distribution of source and target domain samples, so the model trained with source domain samples will not obtain good classification performance for target domain samples. Modelling by continuously acquiring a large number of calibration samples with known labels at different times is a time-consuming and laborious task. In fact, a small number of labeled target domain samples contain knowledge of the target domain sample distribution. Joint modelling with a large number of labeled source domain samples and a small number of labeled target domain samples can discern information about the differences in the distribution of target and source domain data due to sensor drift, thus greatly improving the classification accuracy of the model for the target domain samples. Therefore, acquiring some of the labeled target domain samples at a small additional cost, thereby greatly improving the prediction accuracy of a large amount of unlabeled target domain data, is an acceptable low-cost method to address electronic nose drift.

**Figure 1:**
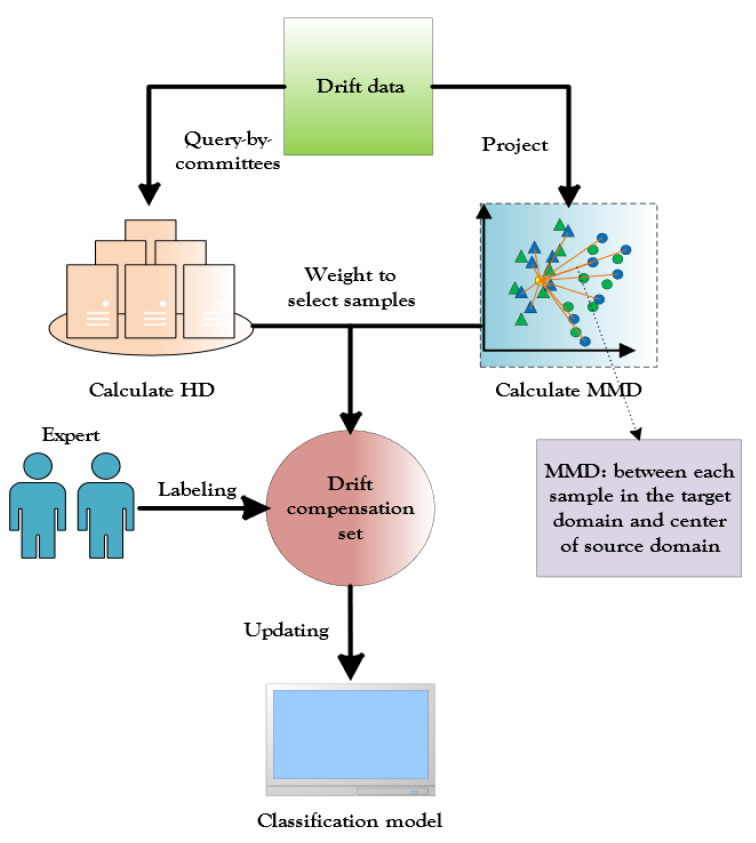
Schematic diagram of the CDAL framework. The cross-domain active learning method using weighted HD and MMD selects samples to update the classification model, which enables more representative labeled samples.

In this study, we aimed to mitigate the effects of distorted data distribution and degraded performance of the E-nose due to sensor drift. We sought to combine an active learning approach and domain adaptation to complementarily enhance the sensor’s performance in the identification of drifting gases. Specifically, we designed a semi-supervised cross-domain active learning (CDAL) model for drift compensation of the E-nose. As shown in Figure 1, for the CDAL method, we used the AL paradigm based on the query by committee (QBC) model to calculate the Hellinger distance (HD) of the distribution of predicted outcomes for each target domain sample committee member, which was used to measure the ease of differentiation of the selected samples. Additionally, we measured the interdomain distribution difference of the data by calculating the maximum mean difference (MMD) of each target domain sample with respect to the source domain center. Finally, we weighted the HD and MMD as the final sample selection criterion, by which the drift compensation samples are selected for the update of the classification model. The new CDAL method considers both the information content of the selected samples and the migration differences and improves the drift compensation performance of the sensor through a combination of domain adaptation and active learning. The method was able to reflect the maximum amount of drift information with a minimum number of labeled samples. To the best of our knowledge, the combination of active learning and domain adaptation has not been used for drift compensation of E-nose sensors.

The advantages of CDAL over other methods can be summarized as follows:

(1) The framework consists of a combination of active learning and domain adaptation, which makes full use of the distribution differences in migration distributions of samples between different domains while considering the degree of disagreement among committee members on the classification results of the sample.
(2) HD is used as a measure of the divergence of the enquiry committee’s output and is more appropriate for sample selection than the usual discrete judgement criteria.
(3) The MMD is able to measure the distributional variability between the source and target domains, greatly facilitating the selection of more representative samples for modification of the classification model in active learning.
(4) The mapping of Gaussian functions allows us to represent the distance between distributions by the inner product of points, which can be used to assess the impact of differences in the distribution of data due to drift.
(5) Experiments show that CDAL is significantly effective in both drift suppression and pattern recognition.

The paper is divided into the following sections: Section 2 reviews related work on active learning. Section 3 introduces the method proposed in this paper, including formula derivation and the optimization process. Section 4 describes the experimental setup, the experimental results, and the sensitivity analysis of the parameters. Finally, Section 5 contains the conclusion of the full text.

## 2. Related Work

### 2.1. Review of Drift Compensation of E-Nose

Due to the influence of the external environment or the ageing of the sensor itself, poisoning and other generated E-nose drift can produce irregular interference with the response of the gas sensor, thus reducing the recognition accuracy of the E-nose system. In recent years, much work has been done to solve the drift problem of E-nose sensors. These methods are roughly divided into three main types: component correction strategies, domain correction strategies, and classifier strategies.

The component correction strategies aim to identify drift components in the original signal and then remove them before training the final discriminative classifier. This approach is primarily based on feature or component removal from sample data. Haugen et al. [7] proposed a mathematical drift compensation algorithm based on corrected samples that maintains the true characteristics of the data and removes sensor drift from the measurement sequence. Regarding drift calibration, most of the current studies in the literature use direct standardization (DS) methods to map the signals from the slave device to the space of the master device to convert the data from the slave device to match the data from the master device [8,9]. Kermit and Tomic [10] proposed a correction model called Independent Component Analysis (ICA), which used higher order statistical methods to analyze gas sensor system data, eliminating the relative components with drift characteristics. Among others, the composition correction method using principal component analysis (CCPCA) attempts to reconstruct the sensor response of a pure gas without drift effects [11]. Ziyatdinov et al. [12] proposed a general PCA method that calculated the drift directions of all classes. In addition to component correction methods, Padilla et al. [13] used orthogonal signal correction (OSC) to find drift elements orthogonal to the label space. As the sensor drift of the E-nose system is nonlinear, irregular, and unpredictable because of the inherent uncontrollability in the sensor manufacturing and use process, we cannot find the exact direction of drift change or there is no fixed direction of drift change at all. Thus, the performances of component correction methods are not satisfactory.

The domain correction strategy is a model that learns from the source domain data distribution and yields good performance on different (but related) target domain data distributions and is well suited for use as drift compensation for the E-nose. Since E-nose drift is nonlinear and unstable, Tao et al. [14] proposed a kernel transformation method to perform domain correction operations, which improves the consistency of the distribution between the source and target domains. Zhang et al. [5] proposed a domain regularized component analysis (DRCA) method to project the source domain data and the target domain drift data, compensating for the drift by making the distributions of the two projected subspaces similar. Zhang et al. also proposed a cross-domain discriminative subspace learning (CDSL) method that achieves drift compensation while ensuring data integrity [15]. Tian et al. [16] proposed the local manifold embedding cross-domain subspace learning (LME-CDSL) algorithm, which is a unified subspace learning model combined with manifold learning and domain adaptation. Wang et al. [17] proposed an extreme learning machine (ELM) with discriminative domain reconstruction, which can improve the classification efficiency of the E-nose by differentiating each domain data and learning a domain-invariant space to minimize the distribution differences between different domains. Yi et al. proposed a unified two-layer drift compensation framework to solve the sensor drift problem considering the distribution alignment of different domains in the feature and decision layers [18]. Yan et al. proposed subspace alignment based on an ELM (SAELM) for E-nose drift compensation, which achieves domain alignment by constructing a uniform feature representation space under multiple criteria [19]. For a combination of convolutional neural networks, Zhang et al. proposed a target-domain-free domain adaptation convolutional neural network (TDACNN), which integrates the use of different levels of embedding features, using intermediate features between the two domains for drift compensation [20]. However, domain correction methods usually require finding a common domain invariant subspace. This is an extremely complex design and requires researchers to develop various indexes for domain alignment, which is not easy to implement.

The aim of classifier strategies is to design robust classifiers to achieve a better discriminative output in E-nose drift compensation. In turn, classifier methods are divided into single classifier methods and classifier integration methods. The single classifier approach is a drift compensation model with a specific method using a single classifier as the discriminant model. Zhang et al. [21] proposed a method based on a domain adaptation extreme learning machine (DAELM), in which a labeled sample of a part of the target domain similar to active learning was used as a reference. Ma et al. [22] proposed a weighted domain transfer extreme learning machine which uses clustering of samples as a criterion to select suitably labeled samples and calculates a sensitivity matrix by weighting them to achieve drift compensation using fewer labeled samples. Tian et al. [23] proposed a Gaussian deep belief classification network (GDBCN) for use as E-nose drift compensation, which compensates for sensor drift at the decision level by cascading a DBN-SoftMax classifier layer based on Gaussian–Bernoulli restricted Boltzmann machines. The integrated approach combines the advantages of multiple single classifiers so that the final integrated classifier outperforms any single component classifier and can greatly improve recognition accuracy. Vergara et al. [24] innovatively proposed a weighted ensemble approach for the base classifier support vector machine (SVM) that solves the gas sensor drift problem over long periods of time while achieving high accuracy. Magna et al. [25] proposed an adaptive classifier integration method to improve the performance of fault and drift sensors for prediction by majority voting decisions. Liu et al. [26] proposed a fitting-based dynamic classifier approach for metal oxide gas sensor integration using a dynamic weighted combination of SVM classifiers trained from datasets collected over different time periods to obtain better classification results. A regularized ensemble of classifiers for sensors was proposed by Verma et al. [27] to apply regularization to the weighted integration of classifiers used as drift compensation. Zhao et al. [28] proposed an integrated model of multiple classifiers based on an improved LSTM and SVM, which improved the performance of the classifier to a greater extent. Rehman et al. [29] proposed a multiclassifier tree model with transient features, where each node uses a different classifier group for integrated classification. Compared with the feature level, it is much more difficult to achieve cross-domain adaptation at the decision level, because the design of a robust classifier itself is an arduous task, which often needs to use labeled target domain samples to implement a domain adaptation classifier for drift compensation. However, if the labeled target domain samples are blindly selected, the correct distribution knowledge of the target domain cannot be obtained, resulting in the failure of the design of the cross-domain classifier.

### 2.2. Review of Active Learning

**Figure 2:**
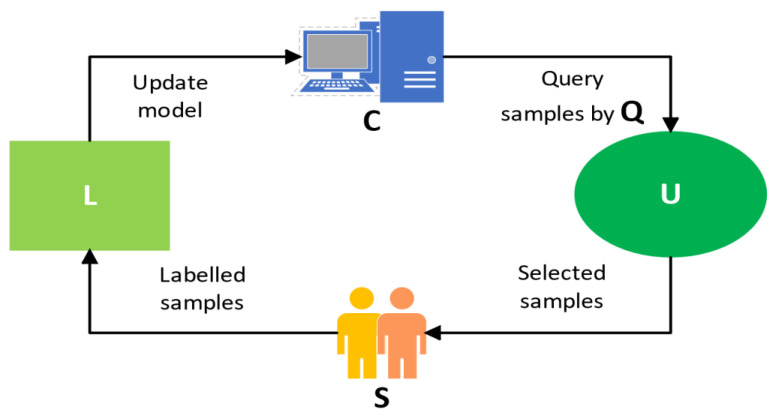
Active Learning Framework.

The AL process is a closed loop consisting of two sample data sets **L** and **U,** which is shown in Figure 2. **U** denotes unlabeled target domain data (sample query set) for drift compensation sample selection, while **L** indicates the drift compensation set (drift compensation samples with labels) for “machine learning model” **C** updating. The “selected samples” **L** are selected from the “query set” **U**, which is full of target domain drifting instances, and the “labels” of the “selected samples” are sent to the “expert” **S** query. The “labels” of the “selected samples” are labeled by “expert” queries. The most critical of these is the “instance selection strategy” **Q**, which requires a suitable rule to select the most representative instances from the data pool for retraining the “machine learning model” **C**. Finally, the “machine learning model” **C** is updated with the selected instances and labels for the next recognition. Therefore, the AL framework is a distinct closed-loop structure that updates the “machine learning model” **C** with “selected instances” **L** and their “labels”, thus improving the model’s recognition accuracy.

In recent years, active learning methods with finite labeled samples have also started to be applied to the drift compensation of E-nose sensors. The core of the AL approach is its “instance selection strategy” **Q**, which aims to reflect the maximum information with the minimum number of samples. Liu et al. proposed an active learning method based on dynamic clustering to balance the labels of different categories of labeled samples by dynamic clustering [30]. Liu et al. also proposed a hybrid kernel-based adaptive active learning approach by designing a hybrid sample evaluation kernel to perform a comprehensive evaluation of the labeled samples [31]. More recently, Li et al. [32] proposed a method combining classifier integration and active learning to reduce the cost of model training by reducing the number of labeled samples and to better suppress the drift of gas sensors. Considering the class-imbalance problem of sample selection, a new metric “classifier state” and an associated sample evaluation procedure are proposed to be used as drift compensation for the E-nose, which successfully reduces the negative impact of the class imbalance problem by using a classifier state sampling strategy [33]. Considering the problem of noisy labeling in active learning, Cao et al. proposed a label evaluation method based on the active learning framework to evaluate and correct noisy labels and improve active learning labeling efficiency [34].

However, AL methods are still in their infancy, and most of the AL frameworks proposed only consider the amount of information contained in the samples while ignoring the effect of misaligned sample data distribution between domains due to drift. In this regard, we proposed the CDAL framework based on the HD and MMD for E-nose drift compensation.

## 3. Methodology

### 3.1. Notations

Suppose $X_{S}=\left[x_{S}^{1},\ldots \ldots ,x_{S}^{N_{S}}\right]\in {ℜ}^{D\times N_{S}}$ denotes the dataset of source domain. *D* represents the number of features and $N_{s}$ denotes the number of samples in source domain. Suppose $C_{S}=\left[c_{S}^{1},\ldots \ldots ,c_{S}^{N_{S}}\right]\in {ℜ}^{D\times N_{S}}$ denotes the label set of the source domain. Similarly, suppose$\;X_{T}=\left[x_{T}^{1},\ldots \ldots ,x_{T}^{N_{T}}\right]\in {ℜ}^{D\times N_{T}}$ denotes the source domain dataset. $N_{T}$ represents the number of samples in the target domain. Suppose $C_{T}=\left[c_{T}^{1},\ldots \ldots ,c_{T}^{N_{S}}\right]\in {ℜ}^{D\times N_{T}}$ denotes the label set of the target domain. ‖·‖ denotes the $L_{2}$-norm.

### 3.2. Cross-Domain Active Learning Approach

Unlike the traditional pool-based AL method, the CDAL method requires the calculation of the information value of the sample and the distribution difference from the source domain from two separate perspectives.

First, we calculated the HD to measure the divergence of the selected samples. For two discrete probability distributions $P=\left\{p_{i},i=1,\ldots \ldots ,n\right\}$, $Q=\left\{q_{i},i=1,\ldots \ldots ,n\right\}$, the HD between them is defined as Equation (1).

(1) $$\;H\left(p,q\right)=\frac{1}{\sqrt{2}}\sqrt{\overset{n}{\underset{i=1}{\sum }}{\left(\sqrt{p_{i}}-\sqrt{q_{i}}\right)}^{2}}\;,$$

For computational purposes, we can also think of this as the Euclidean distance between two vectors of square roots of probability distributions as Equation (2).

(2) $$\;H\left(p,q\right)=\frac{1}{\sqrt{2}}\|\sqrt{P}-{\sqrt{Q}\|}_{2}\;,$$

By definition, the HD is a metric satisfying triangle inequality. The $\sqrt{2}$ in the definition ensures that H(p,q)∈[0,1] for all probability distributions. Considering that we used the QBC method in our framework, we need to calculate the sum of the HD between each pair of committee members as the ultimate disagreement. In this regard, we can obtain a $H\left(x_{T}^{j}\right)$ for each target domain sample$\;x_{T}^{j}\;$as Equation (3).

(3) $$H\left(x_{T}^{j}\right)=\frac{1}{\sqrt{2}}\overset{K}{\underset{j=1}{\sum }}\overset{K}{\underset{h=j}{\sum }}\|\sqrt{P_{j}}-\sqrt{Q_{h}}{\|}_{2},$$

where *K* denotes the number of committee members, and $P_{j}$ and $Q_{h}$ denote the probability distribution of the j-th and h-th committee members for target domain sample $x_{T}^{j}$. As shown in Equation (4), the total number of pairs in the probability distribution is related to the value of *K*.

(4) $$N_{K}=\frac{K\left(K-1\right)}{2},$$

where $N_{K}$ denotes the total number of pairs in the probability distribution. To highlight the variability of the sample when selecting later, we used the sum of the HD obtained by the committee members directly for the calculation. $H\left(x_{T}^{j}\right)\in \left[0,N_{K}\right]$.

We used HD to measure the information value of the selected samples, and next we introduce the MMD to solve the problem of interdomain data distribution differences when selecting samples.

Since we need to calculate the migration distance MMD between each target domain sample and the center of the source domain, the formula for calculating the MMD in this paper is defined as Equation (5).

(5) $$\;\mathrm{MMD}{\left(X_{S},x_{T}^{j}\right)}^{2}=\|\frac{1}{N_{S}}\overset{N_{S}}{\underset{i=1}{\sum }}\varphi \left(x_{S}^{i}\right)-\varphi \left(x_{T}^{j}\right){\|}_{2}^{2},$$

where φ(·) denotes the mapping function.

The key to calculating the MMD is to find a suitable mapping function φ(·) that can map the sample space to a higher dimensional feature space. For this, we first expand Equation (5) to Equation (6).

(6) $$\mathrm{MMD}{\left(X_{S},x_{T}^{j}\right)}^{2}=\frac{1}{N_{s}{}^{2}}\overset{N_{s}}{\underset{i=1}{\sum }}\overset{N_{s}}{\underset{i^{\prime }=1}{\sum }}\varphi \left(x_{S}^{i}\right)\varphi {\left(x_{S}^{i^{\prime }}\right)}^{T}-\frac{2}{N_{S}}\;\overset{N_{S}}{\underset{i=1}{\sum }}\varphi \left(x_{S}^{i}\right)\varphi {\left(x_{T}^{j}\right)}^{T}+\varphi \left(x_{T}^{j}\right)\varphi {\left(x_{T}^{j}\right)}^{T},$$

where $\varphi {\left(\cdot \right)}^{T}$represents the transpose after vector mapping.

According to kernel function theory, the inner product of two vectors in a high-dimensional eigenspace can be found from the kernel function in the original space without knowing the mapping function φ(x)φ(y). Therefore, using the kernel function k(x,y), Equation (6) can be transformed into Equation (7).

(7) $$\mathrm{MMD}\left(X_{S},x_{T}^{j}\right)={\left[\frac{1}{N_{s}{}^{2}}\overset{N_{s}}{\underset{i=1}{\sum }}\overset{N_{s}}{\underset{i^{\prime }=1}{\sum }}k\left(x_{S}^{i},x_{S}^{i^{\prime }}\right)-\frac{2}{N_{S}}\overset{N_{s}}{\underset{i=1}{\sum }}k\left(x_{S}^{i},x_{T}^{j}\right)+k\left(x_{T}^{j},x_{T}^{j}\right)\right]}^{\frac{1}{2}},$$

where k(x,y) denotes the kernel function, which refers specifically to the Gaussian kernel function in this paper.

Considering the mapping space dimensionality of the data distribution, we use the Gaussian kernel function as the kernel function for the MMD calculation:

(8) $$\;k\left(x,y\right)=e^{-\frac{\|x-y\|{}^{2}}{2\delta^{2}}},$$

where δ is an adjustable parameter in the Gaussian kernel function.

The larger MMD between the target domain samples and the center of the source domain indicates the larger difference between the two-domain sample distributions. In other words, the larger the MMD is, the more difficult it is to distinguish. Therefore, we need to calculate the MMD between each target domain sample and the source domain to select the instance with the largest MMD for labeling.

The MMD represents the difference in data distribution between the sample and the source domain, and the HD represents the ease of sample differentiation. A larger HD means that the sample is more difficult to classify correctly, which means that the sample drifts more and reflects the target domain information. A larger MMD indicates a greater degree of migration between the target domain data and the center of the source domain. Therefore, we need to consider the impact of HD and MMD on sample selection in combination. We used an adjustable parameter $\omega_{1}$ to compromise between HD and MMD. The weighted sum $Score_{j}$ of the two terms shown as Equation (9) was used as the final criterion for sample selection.

(9) $$\;Score_{j}=\omega_{1}\mathrm{MMD}\left(X_{S},x_{T}^{j}\right)+\left(1-\omega_{1}\right)H\left(x_{T}^{j}\right).$$

Since we needed to maximize both HD and MMD, we aimed to obtain the samples with larger $Score_{j}$ as the drift compensation set. Therefore, we picked the target domain samples with the first *N* maximum weighted sums for labeling based on the pool-based approach and added them to the training set. Then, we removed them from the target domains. This cross-domain active learning mode can select the most representative samples and greatly improve the recognition accuracy of the classifier.

For a more intuitive representation of our model, we summarized and reorganized CDAL in Algorithm 1.
**Algorithm 1** Proposed CDAL Algorithm**Input:** sample query set **U**, training set $\left\{X_{S},C_{S}\right\}$, test set $\left\{X_{T},C_{T}\right\}$. n: number of samples in each query set **U**. N: number of selected samples. **Output:** updated training set $\left\{X_{S_{1}},C_{S_{1}}\right\}$,updated test set $\left\{X_{T_{1}},C_{T_{1}}\right\}$. 1: Initialize **U** = $X_{T}$.
2: **for**
*i* = 1:N
**do**
3: Calculate the HD $H\left(x_{T}^{j}\right)$ for each sample in **U** by the probability distribution of the committee members’ predictions through Equation (3). 4: Calculate the MMD between each sample in the query set **U** and the entire training set $X_{S}$ through Equation (5). 5: Calculate the weighted sum $Score_{j}$ of HD and MMD with optimized weight parameter$\;\omega_{1}\;$through Equation (9). 6: **end for** **7:** Select N samples with the largest $\;Score_{j}$ from **U** as the selected sample set $X_{N}$. 8: Label $X_{N}$ with category $C_{N}\;$by experts. 9: Update $X_{S}$, $C_{S}$: $X_{S_{new}}$←$X_{S}\;\cup$$\;X_{N}$, $C_{S_{new}}\leftarrow C_{S}\cup C_{N}.$ 10: Update $X_{T},\;C_{T}:$ $X_{T_{new}}$←$X_{T}/X_{N}$, $C_{T_{new}}\leftarrow C_{T}/C_{N}.$

**11: Return** training set $\left\{X_{S_{new}},C_{S_{new}}\right\}$, test set $\left\{X_{T_{new}},C_{T_{new}}\right\}$. 

## 4. Experiments and Results

### 4.1. Dataset

We used a public benchmark dataset of the time drift of the gas sensor array from UCSD [24] for the experimental test, which is widely used by researchers in the field of E-noses for drift compensation study. This comprehensive and rich sensor drift dataset was collected over a period of 36 months on a gas delivery platform. The data were recorded by the E-nose system, which contains 16 MOS sensor arrays (four commercial series TGS2600, TGS2602, TGS2610, and TGS2620), recording a total of 13,910 data samples from exposure to six different gas concentrations, including ammonia, acetaldehyde, acetone, ethylene, ethanol, and toluene. Eight features were extracted from each sensor, and accordingly, each observation was a 128-dimensional (16 × 8) vector. The dataset was then divided into 10 batches according to the month based on the time series collected. Detailed information about the datasets is shown in Table 1. Readers interested in the experimental details of obtaining the dataset can refer to Ref. [24] for further details.

**Figure 3:**
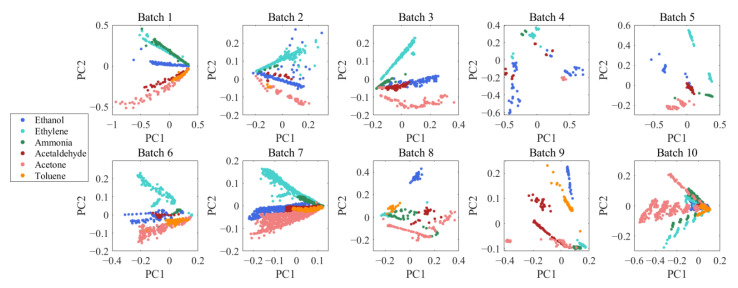
Principal component analysis of ten batches of data. The drift of the data over time batches is evident from the graphs.

To visualize the data drift, we performed principal component analysis on 10 batches of data, mapped the data into a two-dimensional principal into molecular space and plotted the corresponding scatter plots. The PCA for each batch is shown in Figure 3. It is clear that the distribution of the scatter plots plotted from each batch varies considerably, and the reason for this lies in the irregular and time-varying nature of the sensor drift. Therefore, it is reasonable and necessary to compensate for drift from a pattern recognition perspective.

### 4.2. Experimental Setup

To test the compensation effect of our method on sensor drift with the dataset, we took the following two experimental setups:

Setting 1: For the long-term drift of the sensor, we used batch 1 data as drift-free training data, and each remaining batch as drift data for testing.

Setting 2: For the short-term drift of the sensor, we took batch k (k = 1, 2, …, 9) as the training data without drift and batch k + 1 as the test data with drift.

To validate the effectiveness of our CDAL method, we used the QBC method as the active learning query method. For the classifiers, we used the SVM with good classification performance as the chair and the SoftMax classifiers, which can output probabilities, as committee members to evaluate the information of samples. Considering the recognition accuracy, we used the Gaussian kernel for the SVM classifier and performed a grid search for the two parameters c and γ. The range of search for parameter c was [$10^{0}$,$10^{15}$], and the range of search for parameter γ was [$10^{-10},10^{5}$], both set to a step size of $10^{1}$. We initialized the selected sample number N to 30. We set the parameter δ in the Gaussian kernel mapping function to 8, which is calculated by $\delta =\sqrt{D/2}$ based on previous experience. Additionally, we optimized the weighted parameter $\omega_{1}\;$in the interval [0.01,0.99] with a step size of 0.01.

### 4.3. Experimental Results under Setting 1

In Setting 1, we used 12 of the latest drift compensation methods for comparison. First, two single classifiers, SVM and ELM, were used to demonstrate the performance of the classifiers without drift compensation. Note that we used SVM as the baseline classifier for other drift compensation methods. Next, four component correction models, DS [8,9], CCPCA [11], OSC [13], and a generalized least squares weighting (GLSW) [35] method; two typical migration learning methods, DRCA [5] and CDSL [15]; and three methods for picking samples, AL-KLD, AL-JSD, and AL-HD, were compared. The results are shown in Table 2, and the optimal parameters corresponding to the CDAL methods are shown in Table 3.

To make the results more visible, we marked in bold the best results for each batch. Based on the data in Table 2, we can conclude that:

(1) Our CDAL method has the best recognition results among all the compared methods under the same experimental conditions, and the average recognition accuracy is almost 10% higher than all the other methods.
(2) The direct use of SVM and ELM classification was the worst. Among them, the migration learning methods CDSL and DRCA were able to achieve recognition accuracy of 69.59% and 58.72%, which was slightly better than that of the baseline methods. This indicates that the introduction of domain adaptation considering the interdomain distribution problem can improve the E-nose drift compensation effect.
(3) The recognition accuracy of the AL-KLD, AL-JSD, AL-HD, and CDAL methods that used sample selection methods were all above 65%, which was significantly better than the other methods. This also shows that using a small amount of target domain data for labeling can greatly improve the classification accuracy, which is worthwhile and effective.

We also optimized the SVM parameters c and γ and the weighted parameters $\omega_{1}$ involved in Setting 1, and the results are shown in Table 3.

### 4.4. Experimental Results under Setting 2

For the short-term drift pattern of the E-nose (Setting 2), we used the same 13 methods as for Setting 1 as a comparison. From the results in Table 4, we can conclude that:

(1) The CDAL method we used still has the best results in dealing with short-term drift, and with a selected sample of 30, the average recognition accuracy can reach over 82%, which far exceeds the other methods. This indicated that our CDAL method has better identification and robustness than other methods and was very effective in dealing with the short-term drift of the E-nose.
(2) AL-KLD, AL-HD, AL-JSD, and CDAL, as the methods using sample selection, achieved an average recognition accuracy of over 70%, which indicates that selecting an appropriate sample selection criterion for E-nose drift compensation can greatly improve recognition performance. This also shows that the method of obtaining a very small number of target domain labels is worthwhile and effective.
(3) The recognition accuracy of CDSL as a migration learning method can also reach 75.58%, which indicates that in the process of drift compensation of the E-nose system, the difference in data distribution caused by drift needs to be solved, which also provides a reference for our CDAL model.

For short-term drift, we still optimized the SVM parameters c and γ and the weight parameter $\omega_{1}$. The specific optimized values are shown in Table 5.

### 4.5. Parameter Sensitivity Analysis

**Figure 4:**
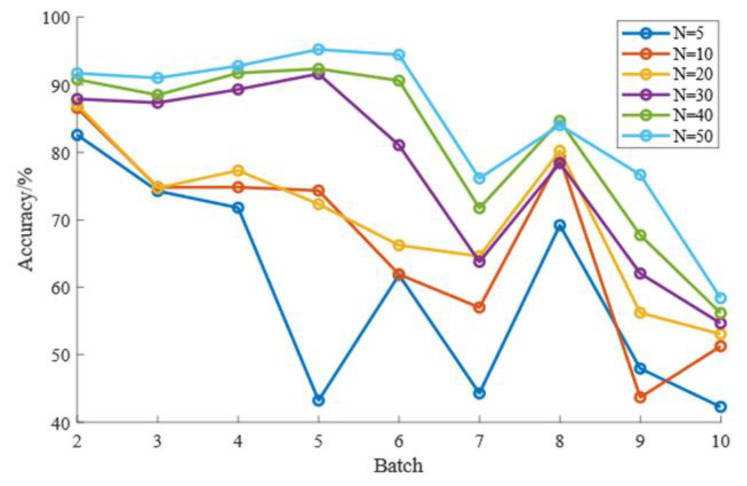
Effect of N on long-term drift.

**Figure 5:**
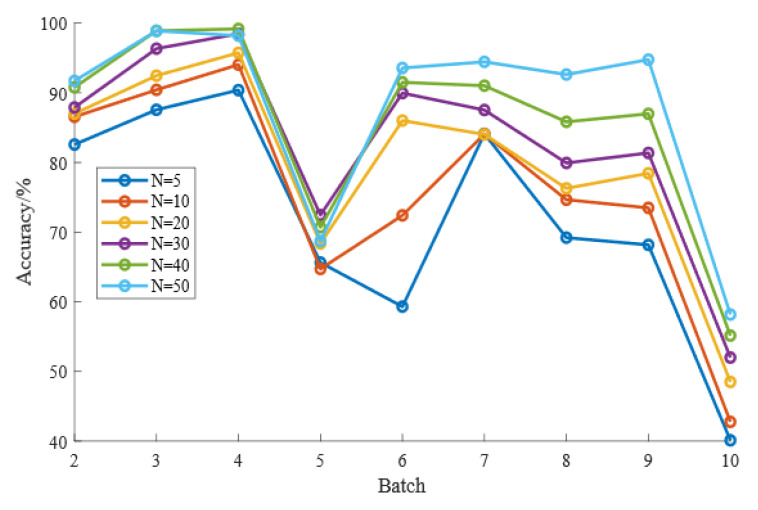
Effect of N on short-term drift.

CDAL requires a weighting rule based on MMD and HD to select samples from the target domain for labeling, so the size of the number of labeled samples, N, can have an impact on the recognition results. It is necessary to observe the effect of the value of N on the effectiveness of the CDAL method. To prevent the effects of overfitting or a small number of markers, we varied the setting of N to {5, 10, 20, 30, 40, 50}. Figure 4 and Table 6 show the effect of the value of N on the long-term drift identification results, and Figure 5 and Table 7 show the effect of the value of N on the short-term drift results.

Based on the data in Table 6 and the visual reflection in Figure 4, we can see that as the number of tagged samples N increases, the long-term drift identification accuracy shows an increasing trend. After the number of picks N reaches 30, due to the small number of samples in some batches, any further increase in the number of markers at this point may cause an overfitting phenomenon, resulting in an insignificant increase in accuracy. Based on Table 7 and Figure 5, we can similarly conclude that the short-term drift compensation effect also becomes significantly better as N increases. Again, due to the small number of samples in some batches, the increase in accuracy is not significant when N reaches 30.

## 5. Conclusions

In this study, we used an approach based on active learning and domain adaptation to solve the sensor drift problem in the E-nose. We proposed a new cross-domain active learning framework based on HD and MMD, called CDAL. We discriminated the amount of information contained in the selected samples by using HD and measured the interdomain distribution differences of the selected samples by using MMD. Finally, we maximized the weighted sum of the two as the selection criterion for the target domain samples and used a pool-based QBC method to obtain the most representative labeled samples. The proposed CDAL method inherits the advantages of active learning, which can greatly improve the classification accuracy by consuming a small labeling cost; additionally, it is the first time that active learning has been combined with domain adaptation, which was also the focus of this study. The ability to introduce the domain adaptation framework into active learning, balancing the impact of interdomain distribution differences on labeled samples, is an important contribution, providing new ideas for future active learning and domain adaptation.

## Figures and Tables

**Table 1:** Brief of drift dataset.

Batch ID	Month	Acetone	Acetaldehyde	Ethanol	Ethylene	Ammonia	Toluene	Total
Batch 1	1–2	90	98	445	30	70	74	445
Batch 2	3–10	164	334	1244	109	532	5	1244
Batch 3	11–13	365	490	1586	240	275	0	586
Batch 4	14–15	64	43	161	30	12	0	161
Batch 5	16	28	40	197	46	63	0	197
Batch 6	17–20	514	574	2300	29	606	467	2300
Batch 7	21	649	662	3613	744	630	568	3613
Batch 8	22–23	30	30	294	33	143	18	294
Batch 9	24,30	61	55	470	75	78	101	470
Batch 10	36	600	600	600	600	600	600	3600

**Table 2:** Comparison of recognition accuracy in long-term drift (%).

Method	1–2	1–3	1–4	1–5	1–6	1–7	1–8	1–9	1–10	Average
SVM	47.99	57.57	65.22	32.99	45.09	35.57	24.83	40.21	31.19	42.30
ELM	69.13	46.22	32.30	46.19	44.91	35.37	25.51	33.19	37.19	41.11
CCPCA	77.65	67.91	65.84	69.54	72.04	54.58	65.31	65.11	37.14	63.90
OSC	79.74	35.25	48.45	52.28	34.30	43.84	49.66	45.32	22.83	45.74
LDA	70.90	73.58	63.35	59.90	63.57	55.58	67.69	47.23	43.22	60.56
DS	42.77	30.90	39.13	48.22	26.35	19.96	48.64	23.19	27.94	34.12
GLSW	72.67	42.37	70.19	52.79	49.78	43.18	57.48	41.91	37.47	51.98
DRCA	64.31	83.35	80.75	74.62	55.04	42.37	48.64	40.00	39.39	58.72
CDSL	79.18	82.85	80.75	76.14	71.78	56.10	74.49	**64.68**	40.31	69.59
AL-KLD	83.63	74.40	62.75	85.63	71.45	53.80	71.78	32.61	54.62	65.63
AL-JSD	75.82	74.42	62.10	83.60	69.82	52.00	72.50	45.00	51.83	65.23
AL-HD	87.16	71.61	62.60	85.18	68.32	50.08	70.88	45.45	47.62	65.43
CDAL	**88.63**	**87.34**	**89.31**	**91.62**	**81.06**	**63.77**	**78.41**	62.05	**54.68**	77.43

**Table 3:** Long-term drift parameter optimization.

	Parameters	Batch 2	Batch 3	Batch 4	Batch 5	Batch 6	Batch 7	Batch 8	Batch 9	Batch 10
Long-term drift	c	$10^{0}$	$10^{3}$	$10^{3}$	$10^{10}$	$10^{15}$	$10^{3}$	$10^{6}$	$10^{6}$	$10^{0}$
	γ	$10^{-1}$	$10^{-1}$	$10^{-3}$	$10^{-6}$	$10^{-8}$	$10^{-1}$	$10^{-5}$	$10^{-7}$	$10^{-2}$
	$\omega_{1}$	0.92	0.98	0.88	0.96	0.86	0.16	0.78	0.13	0.71

**Table 4:** Comparison of recognition accuracy in short-term drift (%).

Method	1–2	2–3	3–4	4–5	5–6	6–7	7–8	8–9	9–10	Average
SVM	47.99	60.03	71.4	58.38	54.69	57.82	69.73	27.02	33.56	53.40
ELM	69.13	63.68	63.98	59.90	47.13	56.02	69.39	26.81	28.69	53.86
CCPCA	77.65	67.15	57.14	55.33	53.26	55.47	75.51	77.45	26.14	60.57
OSC	79.94	73.64	70.19	51.78	56.22	53.67	48.64	61.28	28.89	58.23
LDA	70.90	46.78	82.61	69.04	73.09	56.35	85.71	77.23	16.67	64.26
DS	42.77	43.69	47.83	21.32	28.91	27.35	48.64	16.60	35.58	34.74
GLSW	72.67	66.08	43.48	23.35	27.52	33.63	48.64	68.94	30.58	46.10
DRCA	64.31	66.27	95.03	47.21	54.96	68.92	84.69	72.55	25.25	64.35
CDSL	79.18	77.24	97.52	65.99	74.13	86.44	**89.46**	77.02	34.11	75.58
AL-KLD	83.63	87.68	93.74	70.06	77.43	85.34	75.76	27.02	50.26	72.33
AL-JSD	88.96	93.70	91.60	68.86	71.01	82.19	70.45	74.77	45.52	76.34
AL-HD	86.05	87.52	88.17	70.66	59.52	83.02	73.86	70.57	36.15	72.84
CDAL	**88.63**	**96.34**	**98.47**	**72.46**	**89.96**	**87.52**	79.92	**81.36**	**51.99**	**82.96**

**Table 5:** Short-term drift parameter optimization.

	Parameters	Batch2	Batch3	Batch4	Batch5	Batch6	Batch7	Batch8	Batch9	Batch10
Short-term drift	c	$10^{0}$	$10^{8}$	$10^{6}$	$10^{6}$	$10^{2}$	$10^{10}$	$10^{9}$	$10^{9}$	$10^{7}$
	γ	$10^{-1}$	$10^{-7}$	$10^{-8}$	$10^{-5}$	$10^{-2}$	$10^{-7}$	$10^{-7}$	$10^{-7}$	$10^{-7}$
	$\omega_{1}$	0.92	0.98	0.98	0.40	0.10	0.98	0.97	0.62	0.30

**Table 6:** Classification accuracy with different values of N on Setting 1.

*N*	5	10	20	30	40	50
Batch 2	82.57	86.55	87.01	88.63	90.78	91.71
Batch 3	74.26	74.81	74.71	87.34	88.49	91.01
Batch 4	71.79	74.83	77.30	89.31	91.74	92.79
Batch 5	43.23	74.33	72.32	91.62	92.36	95.24
Batch 6	61.83	61.92	66.23	81.06	90.62	94.44
Batch 7	44.29	57.01	64.60	63.77	71.73	76.14
Batch 8	69.20	79.23	80.22	78.41	84.65	84.02
Batch 9	47.96	43.70	56.22	62.05	67.73	76.67
Batch 10	42.29	51.28	53.07	54.68	56.18	58.39
Average	59.71	67.07	70.19	77.43	81.59	84.49

**Table 7:** Classification accuracy with different values of N on Setting 2.

*N*	5	10	20	30	40	50
Batch 2	82.57	86.55	87.01	88.63	90.78	91.71
Batch 3	87.56	90.42	92.46	96.34	98.90	98.89
Batch 4	90.38	94.04	95.74	98.47	99.17	98.20
Batch 5	65.63	64.71	68.36	72.46	70.70	68.71
Batch 6	59.30	72.40	86.01	89.96	91.50	93.56
Batch 7	84.10	84.07	84.02	87.52	91.02	94.44
Batch 8	69.20	74.65	76.28	79.92	85.83	92.62
Batch 9	68.17	73.48	78.44	81.36	86.98	94.76
Batch 10	40.11	42.76	48.49	51.99	55.14	58.17
Average	71.89	75.90	79.65	82.96	85.56	87.90

## Data Availability

Data is available online at: http://archive.ics.uci.edu/ml/datasets/Gas+Sensor+Array+Drift+Dataset (accessed on 15 June 2022).
